# Structure of Fluoride Anion Aqueous Solution Derived from X‑ray Spectroscopy

**DOI:** 10.1021/acs.jpcb.6c01881

**Published:** 2026-06-08

**Authors:** Vincenzo Carravetta, Johan Söderström, Arnaldo Naves de Brito, Gunnar Öhrwall, Hans Ågren, Jan-Erik Rubensson, Marcus Agåker, Victor Ekholm, Takashi Tokushima, Anirudha Ghosh, Olle Björneholm

**Affiliations:** † 9327 Institute of Chemical and Physical Processes, CNR, Via Giuseppe Moruzzi 1, Pisa 56124, Italy; ‡ Department of Physics and Astronomy, 8097 Uppsala University, P.O. Box 524, Uppsala SE-75120, Sweden; § Institute of Physics Gleb Wataghin, 344101 State University of Campinas, Sérgio Buarque de Holanda, 777 Cidade Universitária ″Zeferino Vaz″ Barão Geraldo, Campinas, São Paulo 13083-859, Brazil; ∥ MAX IV Laboratory, 226073 Lund University, P.O. Box 118, Lund SE-22100, Sweden; ⊥ Faculty of Chemistry, Wroclaw University of Science and Technology, Wybrzeże Stanisława Wyspiańskiego 27, Wrocław PL-50370, Poland

## Abstract

We present X-ray emission (XES) and absorption (XAS) spectra of the F^–^ ion in aqueous solution, along with a theoretical analysis using combined ab initio quantum chemistry spectral calculations and quantum molecular dynamics simulations. The spectra are predicted to have a significantly different dependence on the distance between the anion and the oxygen atoms of water molecules in the first solvation shell. The XES spectrum shows a strong reconfiguration of emission line intensities, while the excitation energy of a resonance in the XAS spectrum is highly sensitive to this distance. Comparisons with the recorded XES and XAS spectra yield predicted F–O distances that agree with 2.7 Å. Two weak features in the XES spectrum are identified as being due to interatomic radiative decay (IRD), thus proving a negative ion counterpart to the recently identified IRD process for the Na^+^ and Mg^2+^ cations in aqueous solution, with the difference that the IRD feature appears on opposite sides of the main ion emission in the two cases. Arguments are provided that the combination of X-ray emission and absorption, together with quantum spectral and molecular dynamics calculations, has much to offer for future studies of the structure and electronic properties of anionic solutions.

## Graphical abstract

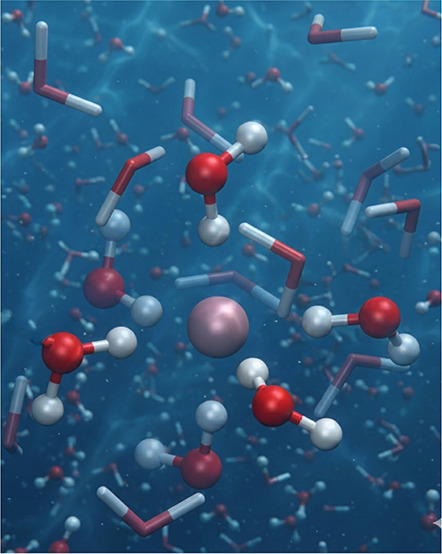

## Introduction

Aqueous solutions containing monatomic ions are ubiquitous in nature and have long been studied for their relevance in several physicochemical and biological processes.^1^ Ions in water have the ability to locally alter the hydrogen bonding network of pure water, to varying degrees depending on their nature, thus altering both its geometric and electronic structure. From a structural perspective, this results in coordination of the water molecules closest to the ion and the formation of solvation shells, where the first one (made up of the water molecules nearest to the ion) is mostly affected as compared to bulk water.

Much of the contemporary interest for halide ions in aqueous solution concerns their effect on water interface properties, so-called salting-in and salting-out effects, with relevance for areas as widely different as atmospheric chemistry and protein physics. Also halide ion bulk solutions have found considerable interest where the effect of the ions has been classified in two categories: structure making (kosmotropic) and structure breaking (chaotropic). Here the smaller ions are believed to strengthen the hydrogen-bond networks due to strong ion–water interactions, while larger ones may disrupt such networks. However, this classification still remains controversial.^2–4^

We recently presented an experimental and theoretical investigation of the radiative decay of Na^+^ and Mg^2+^ in aqueous solutions following ionization of the ion core orbital.^5^ The XES spectrum of these systems shows emission that is energetically separated from the decay from the cation’s 2p orbital (K_α_ band). Theoretical modeling demonstrated that this emission is due to intermolecular radiative decay (IRD) from orbitals that exhibit mixing of the ion’s 2p orbitals with the valence orbitals of water molecules in the first solvation shell. In ref 5 it was highlighted how the IRD process can provide information about the geometric and electronic structure of the water molecules surrounding ions in aqueous solution. With the present report, we intend to extend our investigation on the IRD process to anions, choosing the fluoride ion (F^–^) as a representative case.

Water molecules around F^–^ form a hydration shell due to electrostatic attraction between the negatively charged fluoride ion and the positively charged hydrogen atoms of water. Several investigations, both experimental^6–13^ and computational^4,9,14–22^ have considered the structure of the F^–^ aqueous solution, but the results are still rather scattered. There is general agreement that F^–^ coordinates five to six water molecules in a more or less distorted hexahedral or octahedral structure, respectively.^22^ In such first solvation shell each water molecule points one of its hydrogens toward the ion, while the other one is directed toward the other solvent molecules. The average distance F^–^–H is rather short ∼1.7 Å and the average angle F^–^–H–O is close to 180°.

Due to the F^–^ high polarizability and proton affinity, the F^–^–H hydrogen bond is particularly strong, stronger than that between two water molecules, with a covalent-like character. The structure of the first solvation shell of F^–^ in water is rather rigid and stable with a characteristic water residence time, estimated as about 20 ps, much larger than that of pure water.^18,20^

The electronic configuration of the free F^–^ ion is neon-like, *1s* ^*2*^ *2s* ^*2*^ *2p* ^*6*^. Studies of F^–^ in water using liquid-jet photoelectron spectroscopy have shown that the highest occupied orbital, *2p*, energetically overlaps with the water valence band. Two different values, 8.7 and 9.8 eV, for its binding energy have been reported.^23–25^ Here we present F 1s XAS and XES for F^–^ in water collected by using a liquid jet technique and show how, combined with theoretical ab initio spectral simulations and molecular dynamics (MD) simulations, these provide information about the geometric and electronic structure of anionic aqueous solutions.

## Experiment

Experimental studies of halide ions have employed spectroscopy in different parts of the wavelength spectrum: THz^8,10^ IR, Raman^7,26^ and X-ray absorption,^6,9,27^ mentioning a few examples. The latter X-ray studies did not concern absorption of the halide ions but rather how water X-ray absorption is perturbed in the presence of the ions. Relevant for the present work is also an experimental K-shell X-ray absorption study of hydrated Cl^–^ ions presented by Tongraar et al.^28^ Thus, to the best of our knowledge, there exist no studies of X-ray spectroscopy, neither absorption nor emission, of F^–^ ions in aqueous solution.

### Experimental Methods

The XES measurements were carried out using a cylindrical liquid jet at the VERITAS beamline of the MAX IV synchrotron radiation facility in Lund, Sweden.^29^ The VERITAS beamline is equipped with an elliptically polarizing undulator and a collimated plane-grating monochromator with an ellipsoidal refocusing mirror. It also features a large constant-line-spacing grating Rowland spectrometer, comprising a collimating mirror in the nondispersive direction and a cylindrical grating with 1400 grooves/mm and a 67 m radius.^30^ All measurements were performed with horizontally linear polarized incident radiation.

The sample consisted of 1 M KF solutions, prepared by dissolving high-purity (>98%) KF (Sigma-Aldrich) in Milli-Q water (resistivity 18.2 MΩ·cm). The solutions were delivered into the interaction region via a vertically mounted liquid jet, directed into a liquid-nitrogen-cooled cold trap. The jet was surrounded by a cylindrical differential pumping stage with apertures for the incoming and outgoing photons, enabling intersection of the sample with the X-ray beam in front of the soft X-ray spectrometer. The experimental setup is the same as in ref 5.

Photon emission was detected in the polarization plane of the incident radiation perpendicular to the beam propagation axis. Calibration of the emitted photon energies was based on the K_α_ transition, used to fix the absolute energy scale at a single detector point, combined with elastic peaks to determine the dispersion. Since this procedure depends only on relative energy differences between elastic peaks, it is independent of the absolute energy calibration of the monochromator.

The upper limit of the spectrometer resolution is determined by the total width of the observed K_α_ peak, which is substantially narrower than the fitted widths of the IRD signal components. The X-ray emission spectra were recorded in the first diffraction order.

The vertical binding energy of F 1s (688.8 eV) was measured at the FlexPES beamline of MAX IV, Lund, Sweden.^31^ Photoelectron spectroscopy experiments were performed using a liquid jet with an aqueous solution of 4.0 m (mol/kg solvent) of KF as target. The photon energy used was hν = 750 eV, and the F 1s binding energy was calibrated versus the vertical O 1s binding energy of water (538.1 eV^32^).

#### X-ray Absorption Spectrum

Figure 1 shows a comparison of XAS for the free F^–^ ion,^33^ and F^–^ in aqueous solution. The free F^–^ ion spectrum, is characterized by a broadened step function, without any bound excitation below the core ionization threshold, followed by a cross section that remains almost constant for several tens of eV, as it is expected for the core ionization of an anion that leaves the target in the neutral state. The experimentally obtained core ionization potential of the isolated F^–^ ion is given as ∼681 ± 0.3 eV in ref 33. For F^–^ in water, the vertical binding energy determined by photoemission measurements is 688.8 eV. As is typical for condensed-phase XAS, the general variation of the spectrum in water, compared to that in the gaseous phase, is a rapid decrease in the cross section as the photoelectron energy increases; this gives rise to an apparent maximum just above the ionization threshold. Less expected, is the presence of a well-resolved maximum of the XAS intensity around 694 eV. Something similar was observed in the XAS spectrum of hydrated Cl^–^.^28^ The absence of such peak in the spectrum of the free F^–^ ion suggests that it could be due to interaction with the solvent.

**Figure 1:**
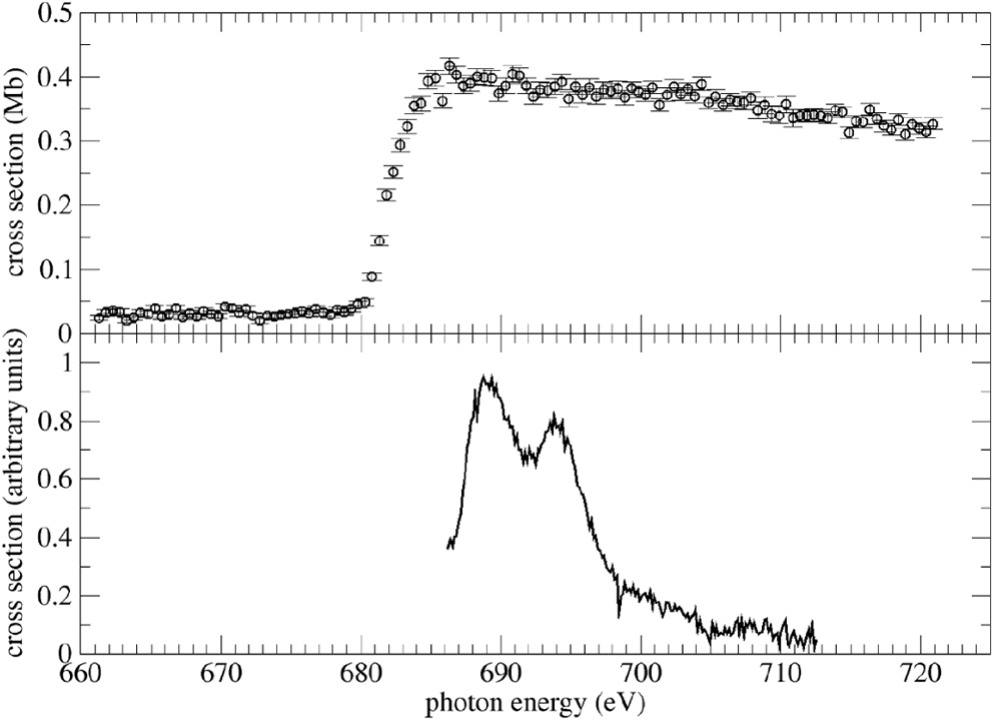
F^–^ K-shell X-ray absorption spectrum; top: free F^–^ in vacuum,^33^ bottom: F^–^ hydrated in water.

#### X-ray Emission Spectrum

Figure 2 shows the measured XES spectrum resulting from the decay of the F^–^ 1 s^–1^ state for hydrated F^–^. The typical low density of free ions does not allow for experimental XES measurements on F^–^ in gas phase, but, as confirmed by the calculations reported in the following, a single K_α_ peak, which is due to the F 1 s^–1^ → F 2p^–1^ + hν radiative decay, can be expected.

**Figure 2:**
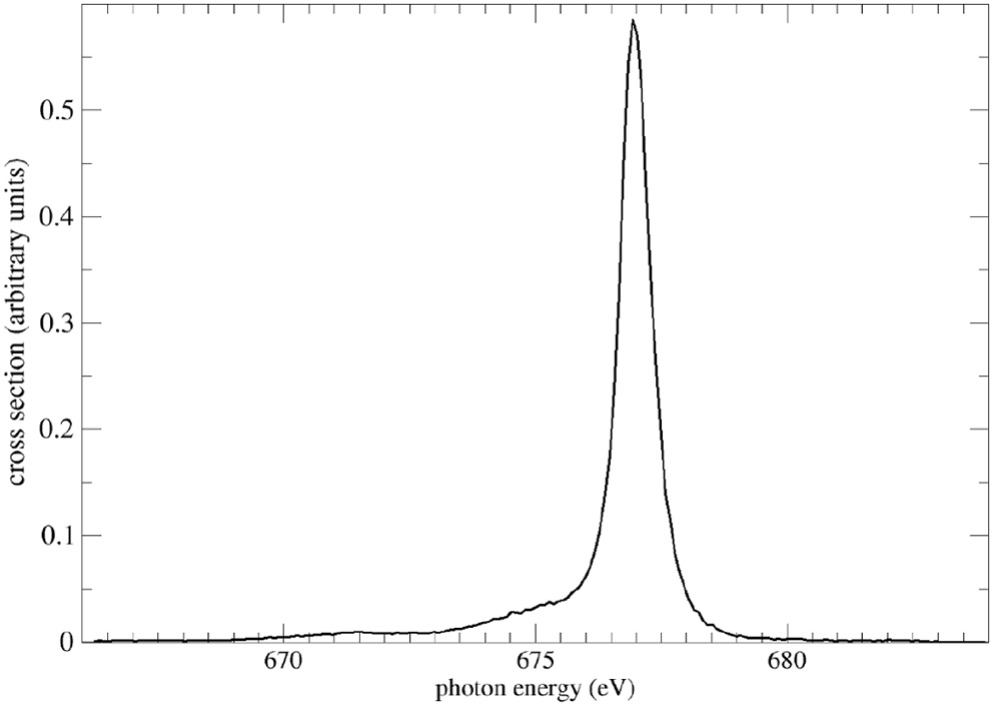
XES of F^–^ in water; experiment recorded using a photon energy of 689 eV.

For the XES spectrum of the hydrated F^–^ ion shown in Figure 2, the 1 s^–1^ state was created using a photon energy of 689 eV, just above the ionization threshold. Other photon energies, covering a range of about 35 eV above the ionization threshold, see SI, were also used which resulted in similar XES spectra, consistent with the emitted electron delocalizing into the water on a time scale shorter than the core-hole lifetime, as observed for Na^+^, Mg^2+^, and Al^3+^ ions in water.^34^ The most intense peak for the hydrated F^–^ ion, at 676.5 eV in the experimental spectrum in Figure 2, is interpreted as due to the local K_α_ decay. On the low-energy side of the K_α_ peak, there are two broad peaks, which are not expected for the case of the free F^–^ ion. These peaks could be due to IRD, as recently observed for Na^+^ and Mg^2+^ ions in water.^5^ The IRD process can schematically be written as

$${F\;1s}^{-1}+H_{2}O\to F^{-}+H_{2}O^{+}{\mathrm{val}}^{-1}+\mathrm{h\nu}$$

where val^–1^ denotes a valence hole on the water molecules in the first solvation shell.

The XES spectra have a larger signal-to-noise (S/N) ratio than the XAS spectra. This difference is mainly related to the experimental conditions required for F K-edge XAS measurements on a liquid jet. The fluorine signal is intrinsically weak compared with the solvent background, and the F K-edge lies in an energy range where the residual continuum contribution from the water O 1s absorption and possible higher-order radiation can introduce an additional slowly varying background. Moreover, XAS scans are more sensitive than XES spectra to point-by-point fluctuations in photon flux, jet position, and surface stability. Importantly, we did not observe evidence that the lower S/N ratio originates from a systematic spectral instability, sample charging, or radiation-induced modification of the sample. Repeated scans reproduced the same spectral shape and energy positions within the experimental uncertainty. The lower S/N therefore affects mostly the visual quality and statistical uncertainty of the XAS spectra, not the main conclusions of the present investigation. The comparison with the calculated spectra relies primarily on robust spectral trends and relative spectral positions, rather than on weak fine details close to the noise level.

## Theory

To assign the measured XAS and XES of F^–^ in water and understand the differences with the measurements for F^–^ in gas phase, we computed both spectra on representative model systems, consisting of small clusters formed by the F^–^ ion and a small number (5 or 6) of water molecules representing the first solvation shell. In fact, our previous similar studies on aqueous solutions of cations (Na^+^, Mg^2+^)^5^ showed, as could be expected, that the radiative decay from core hole states of ions in aqueous solutions is local in nature and essentially involves the water molecules in the immediate vicinity of the ion.

### Computational Methods

Born–Oppenheimer molecular dynamics simulations of the aqueous solution of F^–^ were performed on a sample consisting in one F^–^ anion and 24 water molecules in a cubic box of dimension 9 Å with translational periodicity conditions along x, y, and z. The Quantum Espresso (QE) suite of programs^35,36^ was used with pbe-mt.fhi.UPF pseudopotentials, the PBE density functional and plane waves as basis sets with cutoffs on the wave function and electronic density of 80 and 320 Ry, respectively. The dynamics was performed with a time step of 10 au (0.4838 fs) and extended up to 30 ps. The final part (1 ps) of the resulting trajectory was analyzed to obtain the fluorine–oxygen radial distribution function, see Figure 4. Assuming a limit distance of 3 Å between F and O, all the octahedral structures, i.e., containing the anion and six waters, present in the frames of the final part of the trajectory, were selected. Such structures were then grouped into five different sets containing similar geometries, and for each set the most representative cluster, i.e., the one with the least structural difference compared to all the others, was selected. Such selection was performed using the “cluster” tool of the GROMACS program.^37,38^ By this approach, the structures of five clusters labeled [F^–^6w]_*x*_ with x = a, b, c, d, e were determined. The same procedure was applied to molecular dynamics trajectories where the hexahedral geometry of the first solvation shell was present, in order to select five clusters [F^–^5w]_*x*_. The most representative cluster (x = a) of the set with six water molecules is named [F^–^6w], while for the set with 5 water molecules (x = c) it is named [F^–^5w].

The XES spectrum of any F^–^/water clusters was simulated in the independent particle approximation using the HF wave function with a relaxed hole in the fluorine core orbital as the initial state and the Koopmann’s states with valence holes in the HF wave function of the ground state as the final states. The adoption of such a method, based on the 2-step approximation, might seem questionable in the case of ionizing photon energies close to the ionization threshold. However, its use is presently justified by the experimental observation that the XES spectra collected by us using photon energies up to ∼35 eV above ionization threshold are very similar, as shown in the SI. Calculations have been performed for all the ten clusters mentioned above; the computed XES spectra are reported in the SI. The core BE was calculated by the ΔHF approach fully including electron relaxation, but neglecting both correlation energy and relativistic effects. A larger cluster including 20 waters, see SI, that can be considered as a model of the anion with the first and a second, or more, solvation shell, has been employed to estimate the contribution of the second solvation shell to the BE solvation shift.

The XAS spectrum of the isolated anion and in the presence of water molecules in the first solvation shell was calculated using the Static-Exchange Approximation (STEX).^39^ This is an independent-particle approximation in which the initial state is the HF ground state while the core excited states are obtained from the antisymmetrized product of the relaxed core hole HF state and excited orbitals optimized in the ion field.

The HF calculations were performed with the DALTON program^40^ and the Huz-III basis set of Gaussians, while the STEX calculation was performed with a homemade program interfaced to the DALTON program, projecting the excited orbitals onto a very extended basis of Gaussians capable of describing the electronic continuum. The excitation energies and relative intensities obtained from the STEX calculation form primitive spectra from which, through the Stieltjes Imaging Method (STIM), see for instance^39^ and references therein, the oscillator strength density of the absorption process is obtained with the correct normalization of the electronic continuum.

These methods have been applied to the calculation of XAS and XES spectra of the most representative clusters [F^–^5w] and [F^–^6w] and to a set of four highly symmetric clusters [F^–^ + 6 waters] with a perfect octahedral structure and all F^–^-O distances fixed to a specific value: 2.5, 2.6, 2.7, or 2.8 Å.

### IRD Mechanism for Ions in Water

The IRD process is due to the hybridization of the ion’s p-orbitals with the valence orbitals of the water molecules closest to the ion. Such hybridization can be more or less extensive, giving rise to more or less intense IRD bands, and depends on the relative position of the energy levels of the ion’s p-orbitals and the energy levels that form the valence band of the solvent molecules. It also depends, of course, on the spatial distance between the ion and the water molecules of the first solvation shell. In the case of the Na^+^ and Mg^2+^ cations already studied^5^ the hybridization is rather limited, and IRD gives rise to weak bands localized at higher energies than the K_α_ band generated by the intraatomic radiative decay. In the case of F^–^ in aqueous solution, the IRD process produces a rather different result.

This can be predicted by a simple HF calculation for the ground state of the considered model system. Figure 3 shows, in the left panel, the HF orbital energies of the F^–^ ion and of the water molecule, together with the energy levels of the orbitals of the model cluster formed by the anion and six water molecules. The latter levels form a valence band that covers an energy range slightly larger than that between the 1b_2_ and 1b_1_ levels of the single water molecule. Due to the interaction that occurs in the cluster, both the energy levels of the isolated ion and those of the water band are shifted. The water levels increase and that of the anion decrease. Since the valence levels of an atomic anion are well above those of water, a proximity or almost overlapping of the ion and water band levels of the solvation shell may occur as in the case of F^–^.

**Figure 3:**
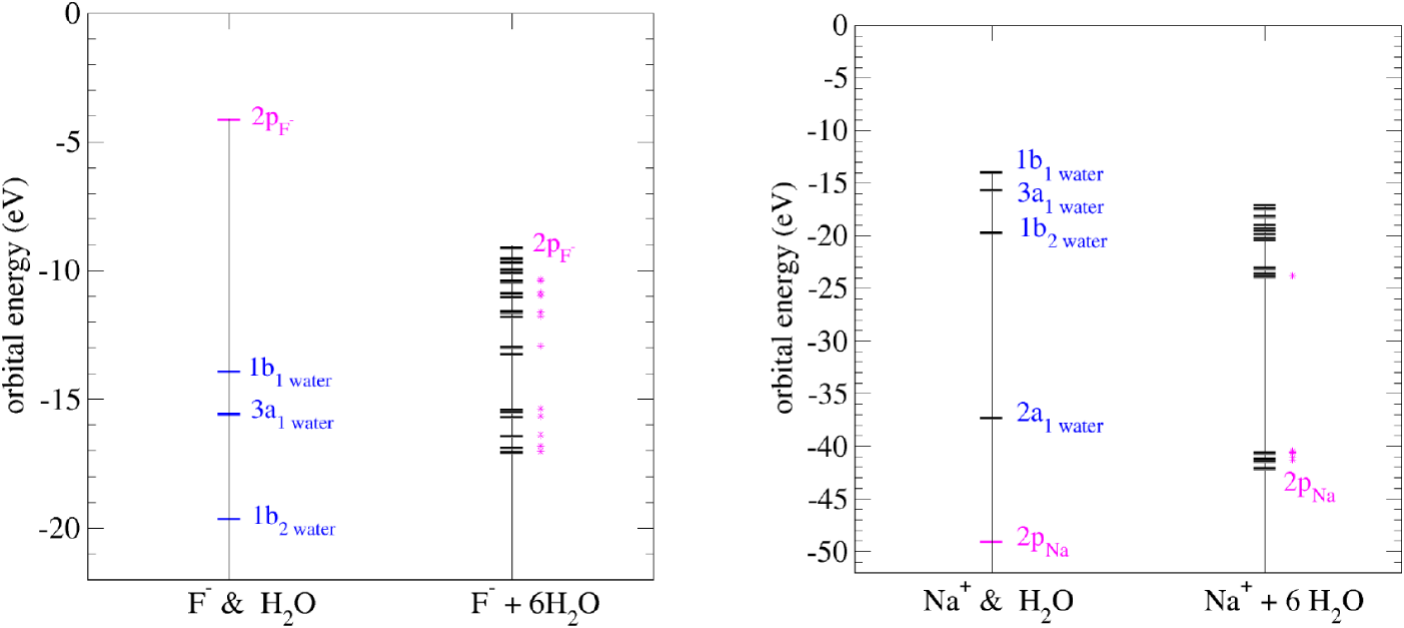
Left panel: HF valence orbital energies of F^–^ and water and of the [F^–^ + 6 waters] cluster. Right panel: HF valence orbital energies of Na^+^ and water and of the [Na^+^ + 6 waters] cluster.

Considering that the intensity of the emission process is dictated by the dipole moment between the ion’s core orbital (highly localized) and a valence orbital of the system, the Mulliken F_2*p*_ population of the different valence orbitals in the interacting system can be taken, as a first approximation, as an index of this intensity. This simple analysis shows that the three 2p orbitals of F^–^ remain identifiable in three orbitals of the cluster, but that a significant trace of them is also present in other orbitals (levels indicated by asterisks in the figure). It is precisely these orbitals that can give rise to IRD bands measurable in the XES spectrum. The position of an IRD band with respect to the intraatomic K_α_ depends on the valence orbital energy, mainly due to the localization of the cluster orbital involved in the decay on the water molecules. Its intensity, instead, is dictated by the more or less large 2p_*ion*_ component of the orbital, i.e., on the partial localization of the cluster orbital on the ion. This is in line with the one-center model for X-ray emission.

It may be interesting to consider the same analysis conducted for Na^+^ in water; the result is shown in the right panel of Figure 3. Unlike the case of F^–^, the interaction in the cluster with Na^+^ leads to a downward energy shift for the valence band levels of water, while the levels related to 2p_*Na*_ increase in energy. The opposite direction of the energy shifts in the case of F^–^ and Na^+^ is simply due to the opposite sign of the two ions and is also true for other cations and anions. Figure 3 also shows how the energies of cluster orbitals related to 2p_*F*_ are widely distributed, whereas those of orbitals related to 2p_*Na*_ are close to each other. In fact, the greater or lesser overlap of the ion and solvent energy levels depends on the electronic structure of the ion. The distribution of the energy levels of the cluster having some ion 2p character depends on the capacity of such 2p orbital to hybridize with the valence orbitals of the surrounding water molecules. The energy levels of the latter, in the absence of interaction with the ion, are at different energy distances from the two ion’s 2p level: approximately 10–15 eV in the case of F^–^ and approximately 30–35 eV in the case of Na^+^. For the cation, the hybridization capacity is therefore more limited and involves a smaller number of valence orbitals of the water molecules.

## Results and Discussion

### Model Systems

Molecular dynamics is a very effective method for simulating the microscopic structure of condensed phases and has also been used for aqueous solutions of halides. However, the characteristic strong F^–^-water interaction is not easily described quantitatively, and numerous computational studies by molecular dynamics at different levels of accuracy have been dedicated to this problem. There is a long and ongoing discussion in the literature about the poor validity for the description of the halide ions -water interaction of standard classical force fields that do not contain terms due to polarization, see for instance^41^ and references therein; but, at the same time, about what value should be attributed to such polarization^21^ for the anion in solution. More sophisticated classical force fields have therefore been proposed, parametrized with high-quality ab initio calculations on model systems. For example, polarizable models based on classical Drude oscillators in conjunction with the polarizable SWM4-NDP water model^42^ have provided results consistent with several experimentally measured thermodynamic properties of fluoride aqueous solutions. Also force fields containing, in addition to a polarizable term for both the anion-water and water–water interactions, a new term that explicitly treats the cooperative bond character of strong hydrogen bond networks^41^ have been adopted. Finally, in recent years, data-driven many-body potential energy functions (PEFs) have been developed which accurately account for higher-order interactions beyond the pairwise approximation, and whose parameters have been optimized with ab initio calculations at the coupled cluster level of theory (CCSD­(T)). A recent paper,^4^ which also presents an extensive comparison of experimental data and theoretical simulation results for the structure of anions in aqueous solution, illustrates the application of these PEFs to the simulation of an aqueous solution of the fluoride ion. All these more recent and accurate classical molecular dynamics simulations confirm the geometry of the first solvation shell predicted by the first and simpler computational investigations but predict a value of about 2.75 Å for the average F^–^-O distance, that is approximately 0.2 Å larger than that provided by the oldest classical molecular dynamics calculations.

In the present investigation, we extended these studies using Born–Oppenheimer quantum molecular dynamics simulations on a system consisting of one F^–^ ion solvated by 24 water molecules, as detailed in the computational section. The system, necessarily small due to the large computational effort of such a calculation, is nevertheless sufficient to provide accurate information on the radial distribution function (RDF) between F and O of water, at least around the first solvation shell, see Figure 4. The value of 2.69 Å obtained for the average F^–^-O distance in the first solvation shell, essentially confirms the result of the most recent classical molecular dynamics calculations conducted with the most accurate classical force fields.

**Figure 4:**
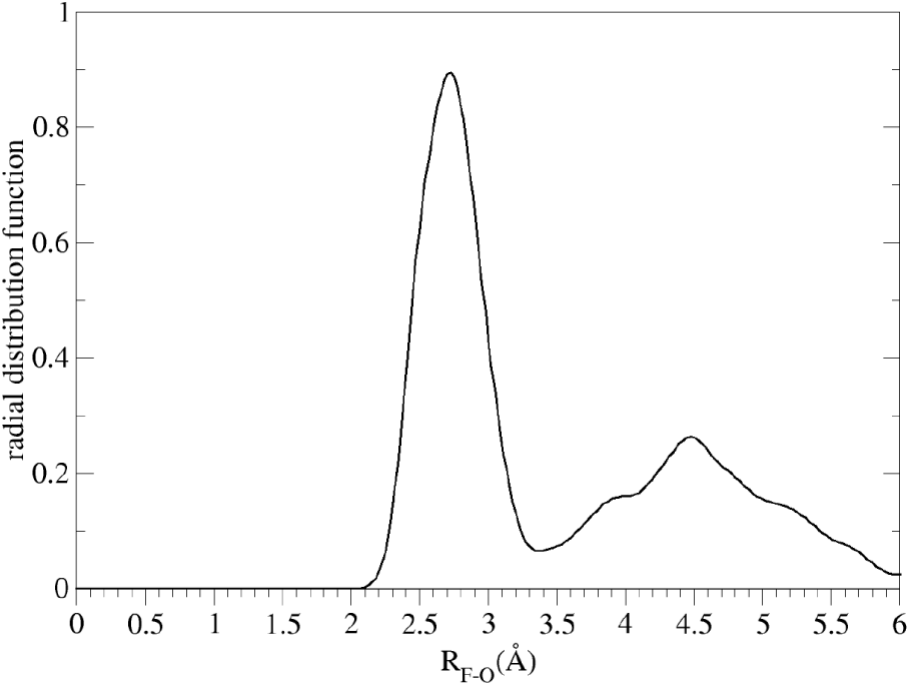
Computed radial distribution function for F^–^ and the oxygen atom of the water molecules.

According to the present quantum molecular dynamics results and in agreement with several previous investigations in the literature, see^22^ and references therein, we have therefore considered either an hexahedral ([F^–^5w]) or an octahedral ([F^–^6w]) cluster, see top panels of Figure 5, as model systems of F^–^ and its first solvation shell, in the simulation of X-ray absorption and emission spectra. The geometry of the clusters was determined by analyzing the trajectories generated by our quantum molecular dynamics, as described in the computational section. The molecular dynamics performed revealed only very rare transitions between clusters with coordination number 5 or 6; the time range (30 ps) investigated is clearly insufficient to describe the coexistence and equilibrium of both structures. The calculations required for this purpose would be very demanding and certainly beyond the scope of the present simulations, which aimed to determine the equilibrated solvation structures considered most probable based on literature.

**Figure 5:**
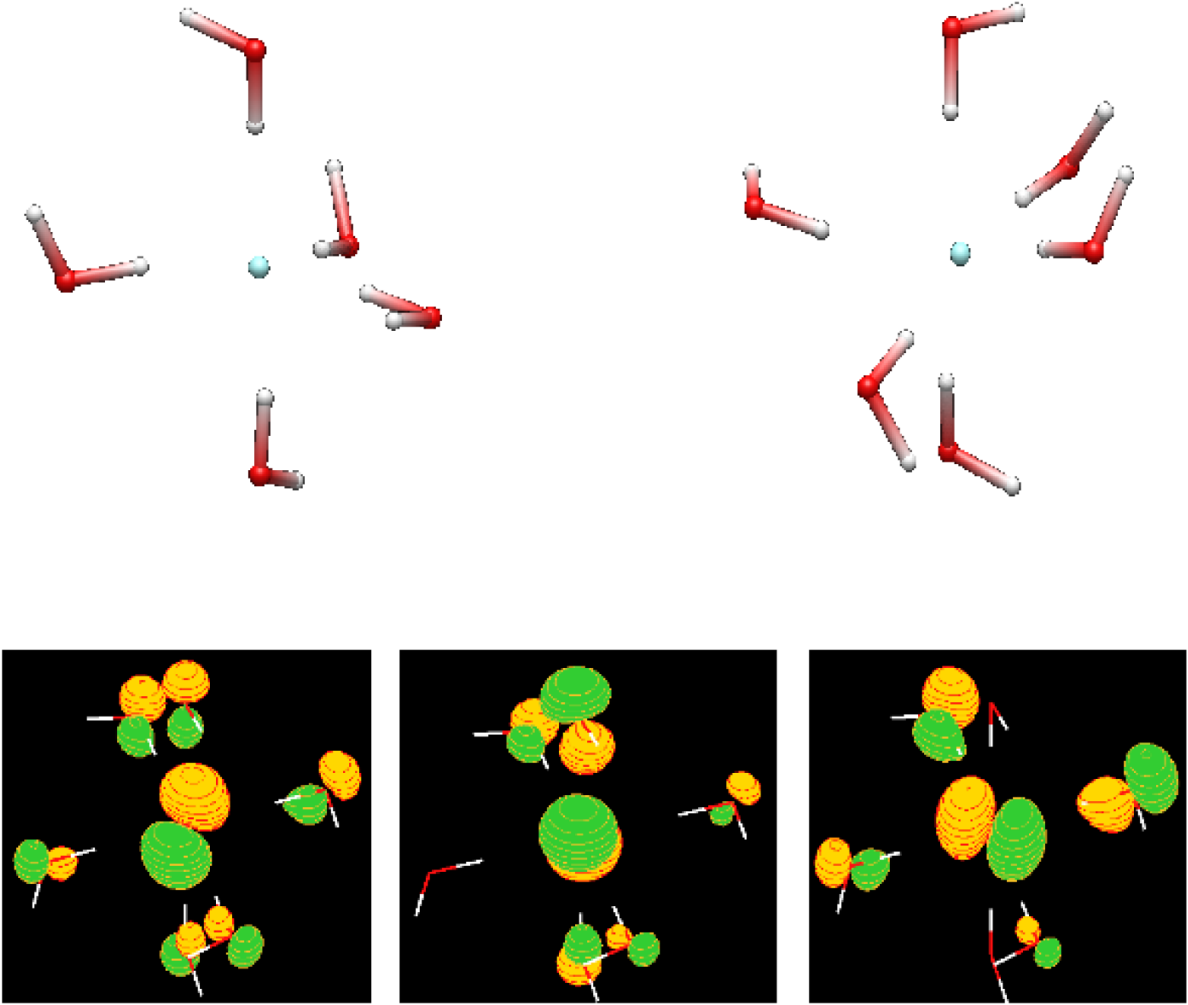
Geometry of clusters [F^–^5w] (top panel—left) and [F^–^6w] (top panel—right) representing the first solvation shell of the anion in the aqueous solution. Fluorine, oxygen and hydrogen atoms are represented by green, red and white balls, respectively. Bottom panels: isosurfaces of the three orbitals of the [F^–^6w] cluster contributing to the IRD band at 671.7 eV of the computed spectrum in the top-right panel of Figure 6. Red (oxygen) and white (hydrogen) sticks represent the water molecules.

### Spectra Simulations

In the left panels of Figure 6 the XAS spectra calculated for the two clusters [F^–^6w] and [F^–^5w] representing the anion in its first solvation shell are compared.

**Figure 6:**
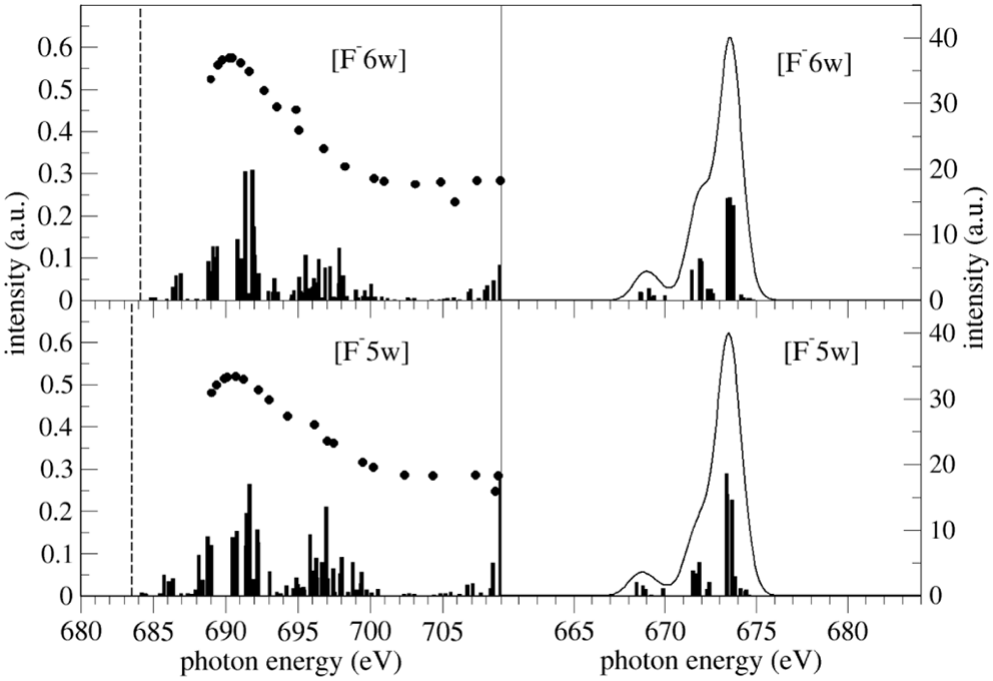
Computed XAS (left panels) and XES (right panels) of: cluster [F^–^6w] (top panels) and cluster [F^–^5w] (bottom panels) representing the first solvation shell of the anion in the aqueous solution. The dashed line in the left panels indicates the ionization threshold, the bars indicate the primitive STEX spectrum, while the dots represent the Stieltjes imaged values of the oscillator strength density. The bars in the right panels indicate the HF spectrum, while the line represent the spectrum convoluted with a Gaussian function (fwhm = 1.5 eV).

The bar spectrum describes the STEX excitations due to transitions from the F^–^ core orbital to the continuum orbitals representable with the discrete basis set. For both clusters the STEX calculation does not highlight any transition to quasi-bound states below the ionization threshold. However, the distribution of excitations in the continuum is rather complex, indicating that the excited states can no longer be described by virtual atomic-like orbitals, but instead by virtual orbitals extended across the water molecules. The mixing of virtual orbitals of F^–^ with virtual orbitals of the waters in the first solvation shell gives rise to a variation of the XAS spectrum of F^–^ when passing from the gas phase to the aqueous solution. A similar mixing, but for the occupied orbitals, will be discussed below to interpret the XES spectrum of F^–^ in water.

The effect of the solvation shell is also appreciable in the variation of the core ionization threshold (dashed lines in left panel of Figure 6). In fact the negative charge of the [F^–^6w] cluster in its ground state, is localized for approximately −0.2 on the water molecules and −0.8 on the F^–^ ion. An immediate consequence of this decrease of electron density on the central atom of the cluster is that the core binding energy is shifted to a higher value than that of the isolated anion due to a reduced electron relaxation around the core hole, as reported in Table 1. The calculated core binding energy for F^–^, [F^–^6w] and [F^–^5w] omits both electron correlation and relativistic effects, that, however, can be considered approximately equal in the three systems. The shift of the core binding energy due to solvation obtained experimentally (7.8 eV) compares to a theoretical value of 5.8/5.2 eV using the [F^–^6w]/[F^–^5w] cluster as model of the solvated ion. This difference is not surprising since a cluster that simply represents the first solvation shell cannot fully account for electronic relaxation and polarization of the solvent. Calculations performed on a larger cluster including 20 waters (see SI), representing the solvent beyond the first solvation shell, show that the computed solvation shift increases by 1.6 eV, so bringing the theoretical prediction to 7.4/6.8 eV in better agreement with the experimental value. In the language of core electron ESCA shifts there are two major contributions to the shifts for ions in solutions—final state polarization which is long-range and initial state electrostatic contributions which is short-range covering essentially only the first solvations shell.^43,44^ The present ab initio calculations intrinsically contain both these contributions and in addition charge transfer.

**Table 1:** Comparison of Experimental and Theoretical Core Ionization Potential of Free and Aqueous F^–^; Δ Values Represent the Solvation Shift

	Experiment	Theory
F^–^ in gas phase^33^	681 ± 0.3	678.4
F^–^ in water	688.8 (Δ = 7.8)	
F^–^6w		684.2 (Δ = 5.8)
F^–^5w		683.6 (Δ = 5.2)

The oscillator strength densities for the two different water clusters [F^–^5w] and [F^–^6w] (black dots in Figure 6) are very similar. Both show a nearly constant trend at high energy and a maximum intensity around 690 eV, i.e., at about 6 eV above the calculated IP, to be compared to an experimental difference of ∼5.2 eV between the ionization threshold and the band observed around 694 eV (see Figure 1). The STEX spectrum (bars in Figure 6 as well as in Figure 7) shows continuum excited states in the energy range immediately above the threshold, despite the known difficulty in their representation by a set of *L* ^2^ functions such as Gaussians. The Stieltjes Imaging procedure, instead, cannot provide reliable values of the oscillator strength density for the low energy electronic continuum, due to its intrinsic low energy resolution.

**Figure 7:**
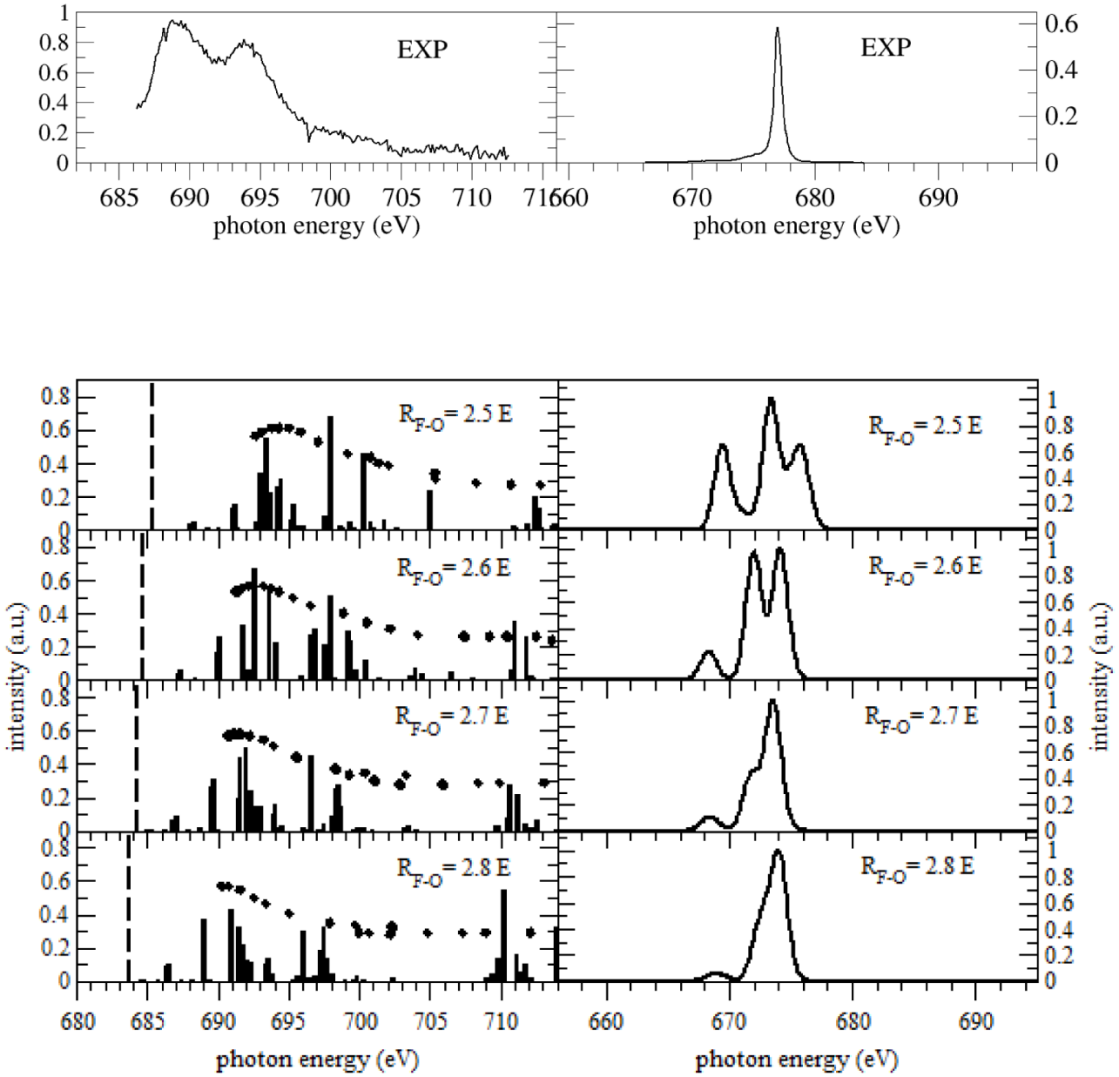
Top: experimental XAS (left panel) and XES (right panel) spectra. Bottom: computed XAS (left panels) and XES (right panels) spectra of ideal octahedral clusters [F^–^ + 6 waters] with F–O distance fixed at 2.5, 2.6, 2.7, or 2.8 Å. The dashed line indicates the ionization threshold, the bars indicate the STEX spectrum, while the dots represent the Stieltjes imaged values of the oscillator strength density.

The XAS band observed at ∼5.2 eV above the ionization threshold can be classified as a “shape resonance”, a term frequently used for similar intensity maxima observed above the core ionization threshold in the XAS spectra of various molecules. The shape resonance is due to the interference produced by the anisotropy of the molecular potential and by multiple scattering of the outgoing photoelectron^45,46^ giving origin to resonant continuum states, that is, continuum states whose localization is increased in the molecular region at the expense of suppression outside that region. Such continuum states cover a specific energy range that is related to the extension of the positive interference region. In a diatomic molecule, for example, such dimension can be associated with the interatomic distance. It has also been suggested^47^ that the existence of shape resonances in a general many-atom system can be considered as conditioned by “barriers” originating from the destructive interference. If the concept of a barrier can be considered questionable in the case of a diatomic molecule, its use does not sound inappropriate in a context like the present one in which a single anion is enclosed in a cage formed by six water molecules. The interatomic distance varies in a diatomic molecule due to molecular vibrations and this contributes to the relatively large width of the shape resonance. It should be observed that for the ion in solution the range of variation for its distance from the first solvation shell can be as large as ∼0.4 Å, because the atomic attraction is weaker than that of a chemical bond.

This interpretation is confirmed by the positional dependence of the here discussed shape resonance estimated by calculating the XAS spectrum for a set of model clusters. Such ideal models have an octahedral structure like the one illustrated in the top-right of Figure 5 but with all F–O distances fixed to a specific value: 2.5, 2.6, 2.7, or 2.8 Å, i.e., in the range suggested by the radial distribution function for F–O in Figure 4. The left panels at the bottom of Figure 7 collect the four calculated XAS spectra; it can be noted how the energy distance between ionization threshold and shape resonance decreases as the R_*F–O*_ distance increases. This is in agreement with the picture in which a resonant continuum has a wavelength proportional to the amplitude R of the molecular region where the constructive interference occurs. Approximating, rather roughly, the continuum by a plane wave with wavevector of magnitude k, the first resonance condition is realized when kR ≈ π. In this case, in fact, the shape of the resonant continuum wave inside the molecular region mimic the lowest stationary state for an electron in a spherical potential well. The continuum energy for the 694 eV band of the experimental XAS spectrum in Figure 1 can be estimated as ≈5.2 (±1) eV, which corresponds to a k value of ∼1.17 (±0.1)­(Å^–1^) and hence an R value of ∼2.7 (±0.2) Å, in good agreement with the average F–O distance predicted by molecular dynamics.

To interpret the XES experimental measurements, we used an HF simulation of the spectrum as described in the computational section for the two model systems [F^–^6w] and [F^–^5w]. The two spectra are reported in the right panels of Figure 6 as bar diagrams and also convoluted (full line) with a Gaussian of fwhm = 1.5 eV for an easier comparison with the experimental profile. An intense band around 673.6 eV is observed, which can be assigned to the intra-atomic K_α_ decay (2p → 1*s*) from the 2p orbitals of the fluorine atom split by the interaction with the water molecules. This band is accompanied by two peaks on the low energy side; the more intense one appears as a shoulder at 671.7 eV, while the weaker one is well isolated at 668.5 eV. Both peaks can be interpreted as due to IRD, based on the electron population and on the shape of the electron density of the orbitals involved; see bottom panels of Figure 5 for the cluster orbitals from which the band at 671.7 eV originates. Such orbitals appear to consist of a mixing between fluorine 2p orbitals and valence orbitals of water molecules in the first solvation shell. The contribution of the water molecules appears as an electron density distributed along the OH bond pointing toward the anion. This density can be seen as due to the combination of the 3a_1_ and 1b_2_ orbitals of each individual water molecule interacting with F^–^. The mixing of ion orbitals and solvent orbitals, which is the basis for the IRD process, has been already observed in our previous study of cations, Na^+^ and Mg^2+^, in aqueous solution.^5^ In the present case of F^–^, the relative position of the valence band of the water molecules and the fluorine 2p level leads to a particularly intense interaction, especially for the IRD band closest to the K_α_ band. The fact that the IRD bands appear, unlike in the case of cations, on the low-energy side with respect to the main band is due to the opposite sign of the ion, as discussed in detail above. It should be added that both the XAS and the XES spectra in Figure 6, computed for the two clusters with 6 or 5 waters are rather similar, suggesting that such spectroscopies could provide little information on the number of waters coordinated by F^–^.

The right panels of Figure 7 collects the XES spectra computed for the ideal octahedral clusters [F^–^ + 6 waters] with a variable F–O distance. It can be noted how, differently from the XAS spectrum, the energy position of the XES bands is quite insensitive to variations of the F–O distance, while the intensity distribution is significantly sensitive. From this point of view, it can be observed that, comparing the spectra calculated with the experimental ones in Figure 7, the best match corresponds to a distance of 2.7 Å, in excellent agreement with what emerges from the molecular dynamics simulation and from the analysis of the XAS spectrum.

For this distance, the values that have been predicted by a number of experimental and theoretical investigations are, in fact, quite scattered. Experimental estimates range from 2.62 to 2.69 Å from X-ray diffraction measurements^11^ to 2.54–2.56 Å from neutron scattering measurements processed with the empirical potential structure refinement (EPSR) method.^12,13^ Similarly, theoretical estimates based on molecular dynamics simulations vary from values around 2.5–2.6 Å using more or less sophisticated classical force fields^4,22^ to values around 2.7 Å using Born–Oppenheimer or Car–Parrinello quantum dynamics.^15,18^ Based on best matching of the here presented XAS and XES experimental spectra with spectra computed by ab-initio methods and on the radial distribution function deriving from present quantum molecular dynamics calculations, we predict 2.7 Å for the F–O distance. Our joined experimental and theoretical study is therefore in favor of the highest values among those present in the literature.

## Conclusions

In this work we have presented the first X-ray emission spectrum of a water solvated anion along with a comparative study of the corresponding X-ray absorption spectrum. The spectra were analyzed with combined ab initio spectral calculations and a Born−Oppenheimer quantum molecular dynamics approach to simulate the solution. Several findings could be unraveled as summarized in the following.

The emission and absorption spectra were theoretically predicted to show quite different behavior with respect to distance between anion and first solvation shell. The X-ray emission spectrum was only minorly shifted in energy but showing a quite strong reconfiguration of emission line intensities, while the X-ray absorption resonance in the continuum turned out to be sensitively dependent on the distance between the ion and the nearby waters. The latter feature bears reminiscence of the shape resonances observed in XAS spectra of small neutral molecules also often showing a remarkable dependence on lengths of bonds that house the corresponding resonance. While distance dependence is sharp, the spectra are predicted to be negligibly dependent on the coordination number of the first solvation shell.

A weak feature in the XES spectrum was interpreted as due to an IRD process, thus a negative ion counterpart to what was recently identified for the Na^+^ and Mg^2+^ cations, indicating a generality of this phenomenon. A difference is that this feature now appears on the low energy side of the main F^–^ emission instead of at the (far) high energy side for the cations. The basis of the IRD process can be traced to the mixing of ion valence orbitals and solvent valence orbitals with an organization of emission energies and intensities that are subtly dependent on both intermolecular distance and molecular orbital energy levels of the free constituents as analyzed in some detail in the present work.

In contrast to neutral systems or cations the core ionization potential of F^–^, like for other anions, shows a positive shift upon an environmental embedding, something that can be referred to the negative charge distribution in the ground state of the hydrated ion. Accounting for the results of a previous solvation model^43^ and the present explicit quantum calculations, we can assign the origin of the shift in terms of polarization, electrostatics and charge transfer, being of that order in magnitude. The water molecules closest to the anion provide the dominant part of all these contributions.

The dependence of the XES and XAS spectra on the first solvation shell distance from the anion is a major result of the present study, while their dependence from the water coordination number is not equally evident. The results of the present investigation indicate that the combination of X-ray emission and absorption together with the use of ab initio quantum spectral and molecular dynamics approaches is a viable path to unravel structure and properties of anionic solutions. More studies are called for to more firmly establish this contention.

## Supplementary Material



## Notes

Figure S1: computed XES of the 5 clusters [F^–^5w]_*x*_ derived from the MD simulation; Figure S2: computed XES of the 5 clusters [F^–^6w]_*x*_ derived from the MD simulation; Figure S3: geometry of the cluster, including one fluoride anion surrounded by 20 water molecules; Figure S4: experimental XES collected using different photon energies (PDF)
