# Supplementary material for: Structure of Fluoride Anion Aqueous Solution Derived from X‑ray Spectroscopy
Source: J Phys Chem B. 2026 Jun 8;130(24):6157–66. doi: 10.1021/acs.jpcb.6c01881 (PMC13288621; doi:10.1021/acs.jpcb.6c01881)
Supplement: Supplementary file 1 [file jp6c01881_si_001.pdf]

SUPPORTING INFORMATION
Structure of fluoride anion aqueous solution derived from X-ray Spectroscopy

Vincenzo Carravetta[1,2], Johan Söderström[2], Arnaldo Naves de Brito[3], Gunnar Öhrwall[4], Hans Ågren[2,5], Jan-Erik Rubensson[2], Marcus Agåker[2], Victor Ekholm[4], Takashi Tokushima[4], Anirudha Ghosh[4], and Olle Björneholm[2]

[1]Institute of Chemical and Physical Processes, CNR, Via Giuseppe Moruzzi 1, 56124 Pisa, Italy

[2]Department of Physics and Astronomy, Uppsala University, P.O. Box 516, SE-751 20 Uppsala, Sweden

[3]Institute of Physics Gleb Wataghin, State University of Campinas, Sérgio Buarque de Holanda, 777 Cidade Universitária "Zeferino Vaz" Barão Geraldo, Campinas - SP, 13083-859, Brazil

[4]MAX IV Laboratory, Lund University, P.O. Box 118, SE-221 00 Lund, Sweden

[5]Faculty of Chemistry, Wroclaw University of Science and Technology, Wybrzeże Stanisława Wyspiańskiego 2750-370 Wrocław PL-50370, Poland

*Email: vincenzo.carravetta@pi.ipcf.cnr.it , Olle.Bjorneholm@physics.uu.se

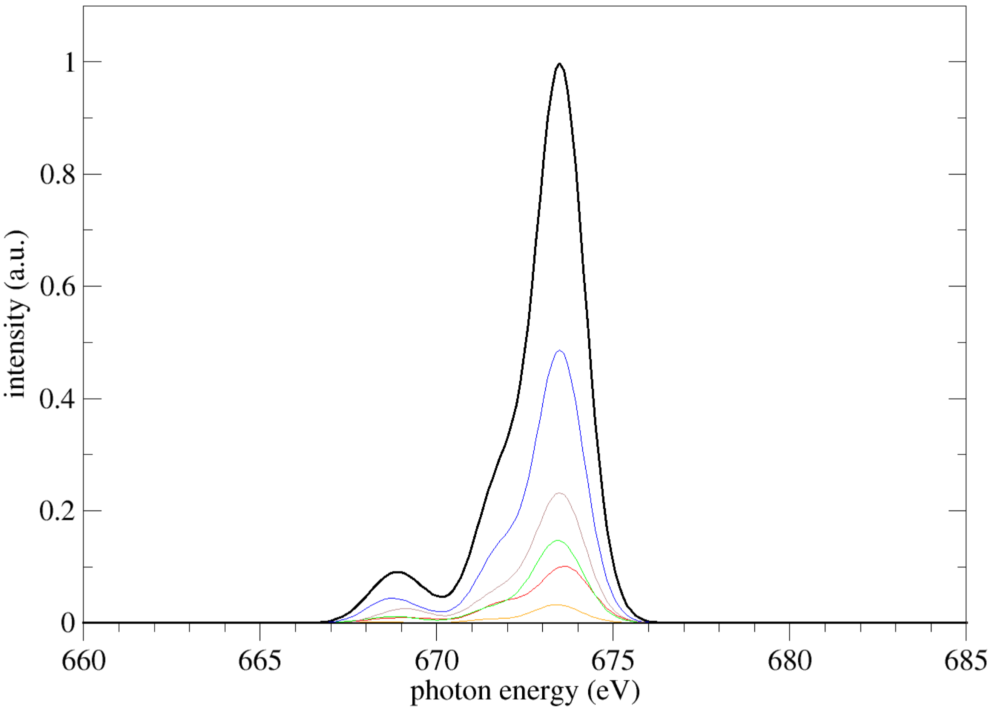

Figure S1: Computed XES of the 5 clusters $[F^{-}5w]_x$ deriving from the MD simulation; x= a(red),b(green),c(blue),d(orange),e(brown). Each color spectrum (is weighted by the percentage presence of the cluster in the sample; the black line represents the averaged XES.

Figures S1 and S2 show the XES spectra computed for a set of 5 clusters containing 5 and 6 water molecules, respectively. Such clusters have been selected from the MD simulations as the ones statistically more representative of the different water geometries for the solvated anion.

Figure S3 shows the geometry of a large cluster describing the distribution of the water molecules beyond the first solvation shell.

Figure S4 shows the XES spectra obtained using photon energy in a range of 0–13 eV above the ionization threshold. Higher photon energies (up to ∼35 eV above ionization threshold, although with lower S/N ratio for the same reason as described below) have also been recorded. All decay spectra are very similar to each other, except for a worse S/N ratio due to the absorption cross section rapidly decreasing with increasing photon energy.

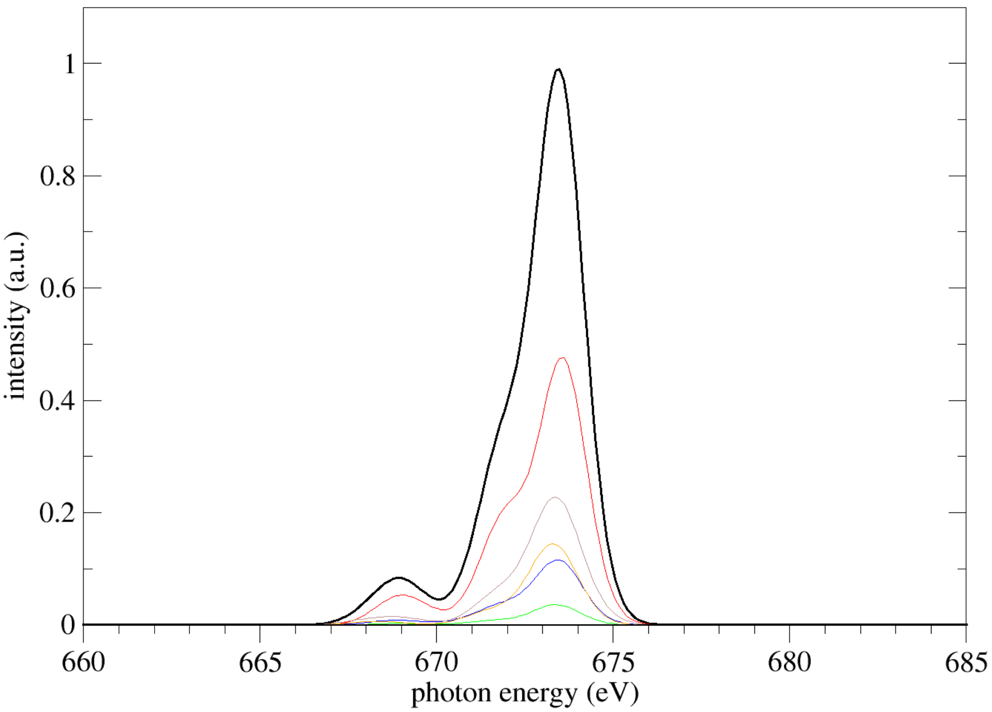

Figure S2: Computed XES of the 5 representative clusters $[F^{-}6w]_x$ deriving from the MD simulation; x= a(red),b(green),c(blue),d(orange),e(brown). Each color spectrum is weighted by the percentage presence of the cluster in the sample; the black line represents the averaged XES.

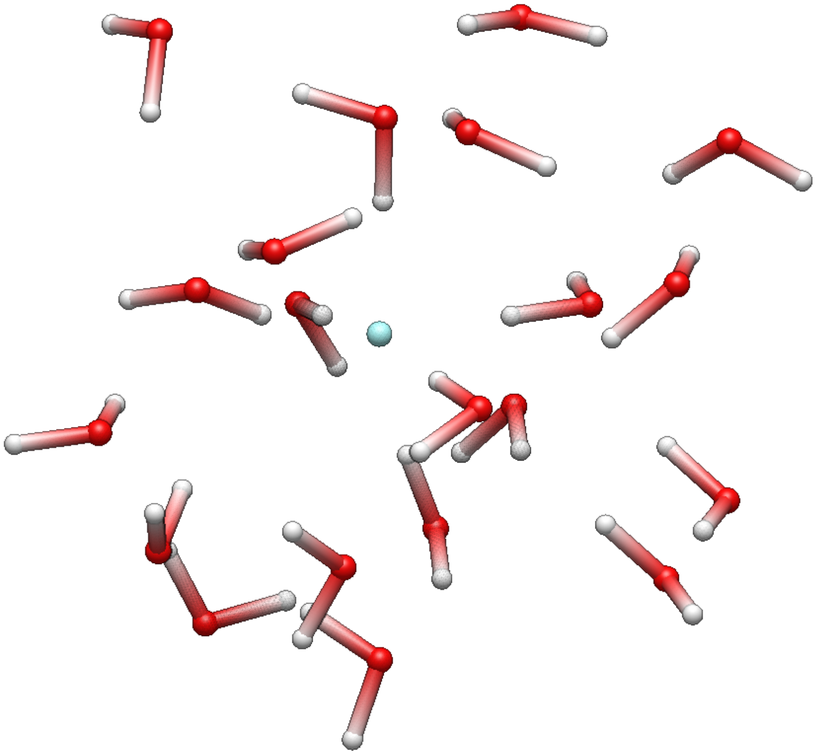

Figure S3: Geometry of a cluster including one F^- anion (green ball) surrounded by 20 water molecules, representing the first and second solvation shell.

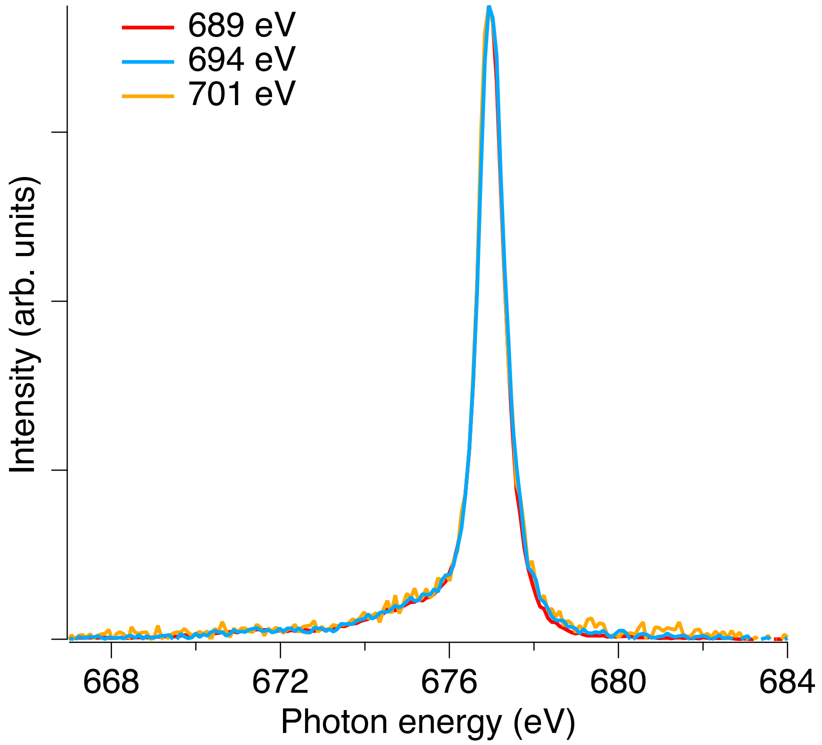

Figure S4: XES spectra collected after irradiation with photons of different energy.
